# Chronic Stress and Oxidative Stress as Common Factors of the Pathogenesis of Depression and Alzheimer’s Disease: The Role of Antioxidants in Prevention and Treatment

**DOI:** 10.3390/antiox10091439

**Published:** 2021-09-09

**Authors:** Gabriela Juszczyk, Joanna Mikulska, Kamila Kasperek, Diana Pietrzak, Weronika Mrozek, Mariola Herbet

**Affiliations:** Chair and Department of Toxicology, Faculty of Pharmacy, Medical University of Lublin, Jaczewskiego 8bStreet, 20-090 Lublin, Poland; gabajul@wp.pl (G.J.); joannamikulska12856@gmail.com (J.M.); kamila.kasperek.pharm@gmail.com (K.K.); dianapchb@gmail.com (D.P.); mrozek.weronika@gmail.com (W.M.)

**Keywords:** chronic stress, oxidative stress, depression, Alzheimer’s disease, antioxidants

## Abstract

There is a growing body of scientific research showing the link between depression and dementia in Alzheimer’s disease (AD). The chronic stress contributes to the formation of oxidative stress in the parts of the brain involved in the development of depression and AD. The scientific literature reports the significant role of antioxidants, which are highly effective in treating these diseases. In this review, we have summarized the relationship between chronic stress, oxidative stress, and the changes in the brain they cause occurring in the brain. Among all the compounds showing antioxidant properties, the most promising results in AD treatment were observed for Vitamin E, coenzyme Q10 (CoQ10), melatonin, polyphenols, curcumin, and selenium. In case of depression treatment, the greatest potential was observed in curcumin, zinc, selenium, vitamin E, and saffron.

## 1. Introduction

Depression is the most common mental disorder affecting more than 264 million people worldwide; almost 800,000 people die due to suicide every year [1,2]. Depressed patients experience anhedonia, fatigue, sleep disturbance, loss of libido, and self-destructive behavior. Depression may be caused by various contributing factors such as, monoamine deficiency, neuroinflammation, neuroplastic changes, and hypothalamic–pituitary–adrenal (HPA) axis hyperactivity [3]. AD is a neurodegenerative disorder, which is the most common form of dementia, resulting in disability of cognitive functions. It is caused by extracellular accumulation of β-amyloid (Aβ) in the form of senile plaques and neurofibrillary tangles. There are two known types of AD: EOAD (early onset Alzheimer disease) and LOAD (late-onset Alzheimer disease). EOAD is a disorder of early onset. It affects people before the age of 65 who are not affected by senile dementia. LOAD is a much more common form of AD. It occurs in patients over 65 years with documented comorbidities.

Epidemiological studies demonstrated that depression is a factor that increases the risk of cognitive decline and development of dementia. These are important symptoms affecting the occurrence of AD in the later years of patient’s life [4]. Both disorders are associated with synaptic degeneration, that may occur in response to increased glucocorticoid level or oligomeric Aβ. It has been demonstrated that these two factors delay exocytosis and therefore affect synaptic functions [5]. Depression has been found both a risk factor and a symptom of AD [6,7,8,9]. People who suffer from depression are twice as likely to suffer from dementia and AD as those who have never suffered from it. It is related to the neurotransmitter system whose disorders lead to neurodegenerative changes. Abnormalities in the serotonin system are already observed in the early stages of AD. Antidepressants acting as selective serotonin reuptake inhibitors (SSRIs) alleviate the impairment of spatial learning and prevent neuronal loss [10].

Stress is defined as a response to potentially threatening stimuli. Although moderate acute stress may be beneficial by enhancing memory performance as well as increasing proliferation of hippocampal cells and neurogenesis, chronic stress may be a cause of deleterious alterations in the brain [11]. Long-lasting stress leads to suppressed cell proliferation and reduced neurogenesis, thus playing an important role in pathogenesis of depression and AD [11]. Environmental stress factors cause symptoms through immune and hormonal effects, neuroplastic changes resulting in neurogenesis, and impairment of neurotransmission [12]. Ultimately, this can lead to neurodegenerative changes and, consequently, to dementia and cognitive decline.

There is an increasing amount of scientific evidence that links depression and dementia in AD; depressive symptoms can occur in up to 50 percent of AD patients [13,14]. Statistical analyses have shown that developing depression may be a predictor of the AD. This is confirmed by meta-analysis, in which the age of AD onset was lower in patients with comorbid depression [15]. The relationship between depression and AD may be due to common etiological action mechanisms. In both depression and AD there may be disorders in noradrenergic transmission [7]. The medial temporal lobe atrophy (MTA) is observed more frequently in AD patients with comorbid depression compared to non-depressed AD patients [14,16]. Moreover, both depression and AD cause inflammation in the nervous system, due to the increased production of similar pro-inflammatory cytokines. Another explanation may be that progressive AD may increase the severity of depressive symptoms. This leads to disturbances in the frontostriatal and frontolimbic pathways [17]. Depression has been shown to increase the neurodegeneration process associated with AD by reducing the volume of the grey matter. In addition, it seems that in both diseases there is microglia activation, which leads to inflammatory processes in the brain. Chronic microglial activation is involved in the progression of the AD. Excessive activation of microglial cells lead to the secretion of some inflammatory markers such as: tumor necrosis factor alpha (TNF-α), transforming growth factor β (TGF-β), interleukin-6 (IL-6), interleukin- 12 (IL-12), or interleukin -18 (IL-18) [18].

The common factor connecting both diseases may also be chronic stress, which leads to oxidative stress. Then, the HPA axis is expanded and the production of glucocorticoids increases. Elevated levels of these hormones can lead to brain damage, especially in the hippocampus. The hippocampus plays a key role in regulating stress in the body. Prolonged exposure to stress has been shown to reduce the volume of the hippocampus and, consequently, to the development of dementia. Chronic inflammation reduces the discharge of monoamines into the synaptic cleft, disrupting the process of serotoninergic and dopaminergic transmission [19,20]. This is further evidence of the relationship between depression and AD. An increased production of Aβ in the cerebral cortex enhances depressive symptoms in the elderly. Moreover, secretion of the glucocorticoids increases the accumulation of the tau protein, which plays a key role in the mechanism of the AD [19]. In both depression and dementia, we observe a disturbance in the brain-derived neurotrophic factor (BDNF) signal pathway [19]. Recent findings on predictors of neurodegenerative disease show that it is possible to prevent some cases of dementia in AD. This confirms that antidepressant treatment is effective in preventing and delaying dementia in patients. This treatment promotes the process of neurogenesis and inhibits the deposition of Aβ in neurons [19,21].

## 2. Chronic Stress and Changes in the Brain

Stress response is mainly regulated by HPA axis. Studies have shown an association between hyperactivity of the HPA axis and chronic stress. Long-lasting stress stimulates the secretion of corticotropin-releasing hormone (CRH), which causes the synthesis and secretion of the adrenocorticotropic hormone (ACTH) in the pituitary gland. ACTH stimulates cortisol production by adrenal glands. Elevated cortisol level causes dysfunction of the centers regulating mood, psychomotor drive, mechanisms of memory and emotionality, disturbances in neurogenesis, and impaired function of hippocampus formation. With increased cortisol secretion, the hippocampus inhibits the secretion of CRH. When this function is impaired, increased secretion of corticosteroids is observed causing damage to the hippocampal neurons [22,23,24]. Both in vivo and in vitro experiments have shown that stress-level glucocorticoid administration elevates Aβ formation and accelerates formation of neurofibrillary tangles, leading to memory impairments [25]. Additionally, cortisol may increase the damaging effects on the brain of Aβ [3]. The hypothesis of depression as a result of HPA axis disturbances is confirmed by dexamethasone suppression test (DST). Dexamethasone inhibits secretion of ACTH by pituitary, resulting in decreased level of plasma cortisol. Dexamethasone nonsuppression suggests impaired feedback regulation and hyperactivity of HPA axis. Although depressed patients have impaired HPA axis function, DST cannot be considered as an indicator of depression. Failed DST may also be observed in patients suffering from other disorders such as AD, eating disorder and bipolar disorder. DST, however, may be associated with severity of depression and risk of relapse [22,25].

Stress caused by various factors lead to the activation of the immune system and increased production of proinflammatory cytokines. Prolonged immune response results in dysregulation of the neuroendocrine system [22,26,27]. The immune reactions in the central nervous system involve microglia consisting of resident macrophages. Prostaglandins, chemokines, and nitric oxide are released from microglia causing inflammation, which is a reliable indicator of the presence of pathogens [26]. Pro-inflammatory chemokines, acute phase reactants, adhesion molecules, and cytokines act on NF-ĸB (nuclear factor kappa-light-chain-enhancer of activated B cells) in the brain, resulting in a decreased amount of monoamines, excitotoxicity, and trophic factors [22,27]. Nitric oxide activates glutamatergic neurons, damaging neurons by nitrosation of nucleic acid [26]. Many studies have shown a direct link between chronic inflammation and sustained activation of the immune system with pathological processes in depression and AD [27,28,29]. In patients with depression and AD raised levels of pro-inflammatory cytokines, acute phase proteins (APPs), interferon gamma (IFN-γ), interleukin 1 (IL-1), IL-6, and TNF-α were observed [3,30,31]. The size of the increase in these factors also determines the severity of symptoms [27,32]. In addition, anti-inflammatory drugs, such as Celecoxib (a selective COX-2 inhibitor, non-steroidal anti-inflammatory drug) and Etanercept (TNF-α antagonist) may alleviate symptoms of depression [22,29]. Antidepressants also decrease IL-1, IL-6, and TNF-α levels and rescued synaptic pathologies [5,29]. Cytokines influence neuroendocrine functions, neuroplasticity, and neurotransmission. IL-1, IL-6, and TNF-α increase CRH production, leading to HPA axis hyperactivity. Interferon α (IFN-α), IFN-γ, and TNF-α up-regulate the serotonin transporter (SERT). Several animal studies have shown that administration of INF-α results in reduced dopamine level in cerebrospinal fluid. Proinflammatory cytokines cause glutamatergic activation, leading to damaging neurons. Increased TNF-α and IL-1 levels have been observed in hippocampus of depressed patients, which may be associated with impaired neurogenesis [22,26].

The hippocampus is a brain structure that plays a key role in the body’s response to psychosocial stress. Neurons found in the hippocampus lead to the expression of glucocorticosteroid receptors. Inhibitory sections in the hippocampus prevent and inhibit the release of CRH. Long-term exposure of the hippocampus to corticotropins causes damage to its cells and impairs cognitive abilities. As a result, the volume of the hippocampus decreases. Structural changes in the hippocampus result in impaired neurogenesis as well as splitting and shortening of the dendrites. Therefore, it has been hypothesized that prolonged exposure to stress and depression leads to structural changes within the hippocampus and thus contributes to the development of dementia [19].

## 3. Chronic Stress in Depression and AD

Stressful situations are a factor that often precedes the occurrence of a major depression episode, due to the increased level of glucocorticoids in the blood. This is a known predisposing factor for both major depressive disorder (MDD) and AD. Patients with current depression or after an episode show an increased sensitivity to cortisol. Cortisol is a model substance used in animal models (mice) to induce depression [3] and a stress hormone. A study performed on the group of 220 elderly women and men (age between 65–83 years) has indicated higher level of markers oxidatively generated DNA and RNA damage, such as 8-oxo-7,8-dihydro-2′-deoxyguanosine and 8-oxo-7,8-dihydroguanosine in older people urine. The obtained results show that there is a connection between cortisol excretion and oxidation markers. The mentioned study supports the thesis that stress, aging process, and occurrence of neurodegenerative diseases are connected to chronical higher cortisol level in blood [33]. Moreover, as reported in the literature, MDD decreases the volume of the hippocampus. Hypercortisolism, a result of Cushing’s disease, reduces the volume of both the hippocampus and the frontal lobe, with apparent white matter atrophy, the consequence of which is cognitive decline long after disease remission. This suggests a possible connection between inflammation, oxidative stress, and depression episodes. Although it remains unclear whether the decrease in hippocampal volume is a cause or a consequence of depression, it is known that the greater the decrease in the volume of this part of the brain is, the longer the expected duration of a depressive episode and the risk of relapse. Additionally, a decrease in the size of the hippocampus may indicate a predisposition to the development of subsequent mental and degenerative diseases, which would indicate a possible correlation between AD and MDD [5]. Chronic stress reduces the efficiency of the immune system, contributes to carcinogenesis and increases a risk of developing cardiovascular diseases. Stress-related mental illness, such as depression, is considered to be associated with the increased non-suicide mortality [33]. Furthermore, it has been proven that treatment with glucocorticoids has a negative influence on the course of treatment of a posttraumatic brain injury [3]. Scientist agree that chronic concentration of glucocorticoids, in contrast to acute instant cortisol release, has a negative impact on the brain’s tissue. This suggest that chronic stress, as a factor of increased secretion adrenal cortex hormones, may be correlated with an episode of depression, and in older age—also with neurodegenerative changes in the brain and AD [34,35]. However, it has to be mentioned that the optimal level of this hormone is individually differentiated and subject to fluctuations in the circadian rhythm and psychological circumstances. The problem is that, relying only on a blood test, it is impossible to define whether the patient has an optimum or an excess of cortisol concentration. Significantly, in blood patients suffering from MDD there is chronic glucocorticoids overflow and incorrect fluctuations depending on the time of the day. This predisposes one to develop AD in old age. Due to the increased concentration of cortisol with age, it is difficult to clearly say whether this increase is related to aging or correlated with AD. In the brains of these patients a pathological variant of Tau and Aβ proteins were observed. In the course of an experiment on mouse models, inducing stress or chronic administration of glucocorticoids has accelerated formation of deposits of these pathological proteins [36,37]. In addition, when mifepristone (glucocorticoid receptor antagonist) was administered to mice, the formation of Aβ and tau protein was inhibited. This suggests that the increased cortisol level may enhance the neurodegenerative cascade, and thus contribute to the AD onset [38].

## 4. Chronic Stress—Oxidative Stress

Research confirms that chronic stress contributes to the development of oxidative stress in the parts of the brain involved in the development of depression and AD [39]. The disturbance in the balance between antioxidant defense and the free radicals’ (ROS) production is defined as oxidative stress [40]. Biological systems in a process of metabolism generate products whose overproduction may be harmful to the whole human organism. In normal condition, the amount of radicals and antioxidants should be aligned. Oxidative stress disturbs redox signaling and controlling, which leads to biomolecule damage [41]. Superoxide radical, hydrogen peroxide, hydroxyl radical, and singlet oxygen are commonly defined reactive oxygen species (ROS) [42]. The reactive nitrogen species (RNS) is a group of molecules derived from nitric oxide and superoxide. Nitric oxide (NO), nitrate (NO_3_^−^), and nitrite (NO_2_^−^) are several factors which play a direct role in cellular signaling, vasodilation and immune response. RNS act together with ROS in cell damage [42]. Multiple metabolism processes, like apoptosis or proteins phosphorylation, need a proper ROS production in the cells, which must be kept at a low level. The ATP (the main form of energy in cells) is generated at the mitochondrial inner membrane. The same area is also the location of the main source of ROS- electron transport chain (ETC) [43]. The major amount of undesirable ROS/RNS are from leaking mitochondrial membranes and from immune cells combating antigens. ROS play an important role in redox biology in homeostasis, cell-signaling but also are responsible for the response of the immune system to violating microorganisms. In addition, ROS and RNS regulate numerous biological processes such as: energy metabolism, blood circulation, embryonic development, reproduction, and remodeling through apoptotic mechanisms in both the cells and tissues [41].

Overproduction of ROS begins to show destructive effects on significant cellular proteins, nucleic acids, and lipids. Biomembranes are attacked by excess of radicals and this propagates lipid peroxidation chain reaction, which leads to various types of cell death: apoptosis, autophagy, and ferroptosis. Ferroptosis is a type of iron-dependent programmed cell destruction. Through accumulation of iron, the glutathione-dependent antioxidant defense fails, which leads to massive lipids peroxidation and cell death as a consequence. In the newest studies, ferroptosis has been linked to diverse brain disorders [44]. Ferroptosis is contributed to intracerebral hemorrhage, whose effect is neuronal death [45,46]. During the neurons damage caused by ferroptosis, lipid metabolites from inside the cell body are released. These noxious metabolites destroy surrounding neuronal cells and indicate that there is an inflammation process inside the brain. As a consequence, some neurologic diseases like AD, Parkinson’s disease (PD), and ischemic and hemorrhagic stroke may appear [47]. The evidence that ferroptosis has influence on the onset of AD can be found in a study performed on animal model. It has been found that lack of glutathione peroxidase 4 (Gpx4) enzyme intensifies the activity of ferroptosis. Vitamin E promotes Gpx4 activity, consequently preventing inflammation in the brain and also inhibiting ferroptosis. It was observed that the mice with decreased Gpx4 levels suffer from cognitive impairment and neurodegenerative changes in the hippocampus [44]. A specific transcription factor, ATF-4, indicates cells susceptibility to death. The presence of this protein impacts the neurons’ resistance potential against ferroptosis; however, its excess promotes neurodegeneration [48,49]. Aβ is a toxic peptide whose presence in post mortem brains is generally specific for AD patients. This substance is created as a result of free radical activity and consequently becomes a factor of the onset and progression observed in AD [42]. On the other hand, there is a concept known as mitohormesis. It has been proposed that the proper amount of ROS may cause protective responses that promote body cells to better manage stress in the future [50].

Nearly all stressors have the ability to activate both sympathetic nervous system and HPA axis. Axis activation causes ejection of ACTH, which stimulates adrenal glands to secretion of catecholamines and corticoids, including cortisol. The consequence of this is the onset of inflammation and oxidative stress. It may suggest that a hypothetical way of prevention this condition is the use of antioxidants [36,51]. Corticosteroids are the class of steroid hormones produced in the adrenal cortex in adrenal gland. One of them is cortisol, which is released in a diurnal cycle. Cortisol is called a stress hormone, since its release is increased during the stress reaction. Prolonged secretion of cortisol may play a critical role in damaging mechanisms of chronical psychological stress. The excessive amount of cortisol contributes to the release of free radicals, the excess of which generates oxidative stress. This process may damage the structures of DNA or RNA and influence the aging process [36]. There is plentiful evidence that suggests the excess of corticosteroids and chronic stress have a harmful influence on human brain; it may directly or indirectly damage cerebral tissue. The indirect path relies on increasing the effect of Aβ, which is a known agent found at postmortem examinations in the brains of people suffering from AD [7,36].

## 5. Oxidative Stress in Depression and AD

It has been proven that oxidative stress accelerates aging of the body. Moreover, it leads to development of many diseases such as cardiovascular disease, diabetes, or cancer. It is caused by excessive production of ROS and free radicals. The molecular mechanism of oxidative stress involves DNA damage, genetic mutations, changes in functioning of proteins, and the induction of apoptosis. Several studies have shown that oxidative stress is also the cause of diseases such as depression or AD [52,53]. Meta-analyses show a correlation between the increased levels of oxidative stress biomarkers and development of depression [54].

The factors contributing to the development of depression are not fully understood. Research indicates a relationship between oxidative stress and inflammatory processes and the occurrence of depressive disorders. In a stressful situation, there is an increased secretion of ROS. As a result of oxidative stress, ATP levels are reduced, leading to inhibition of glycolysis. ATP is required for the first step of glycolysis, where glucose is converted to fructose-1,6-biphosphate. In this step, two ATP molecules are used for each sugar molecule. The concentration of glutathione, which is a strong antioxidant, also decreases [55]. There is also an increase in calcium concentration in the cytoplasm due to inactivation of the calcium pump, increased membrane permeability due to membrane depolarization and oxidative damage to DNA. The production of ROS leads to the secretion of inflammatory mediators, especially prostaglandins and leukotrienes, which in turn can lead to neurodegenerative changes in the brain. Chronic exposure to stress disrupts the prooxidant–antioxidant balance towards oxidative reactions. ROS result in the formation of unusual free radical oxidation products that react with cellular metabolites. This may result in cell death by apoptosis or necrosis [55,56,57]. The main source of ROS production is the mitochondrial respiratory chain. Due to its high level of energy consumption, the brain is highly dependent on mitochondrial activity [56,57]. A significant number of patients complain of persistent side effects after taking antidepressants, most commonly: “feeling foggy or detached, drowsiness, reduction in positive feelings, thoughts of suicide, addiction” [58]. In addition, many patients are refractory to currently used drugs. Therefore, the understanding of pathomechanism of the disease becomes an important factor that could play a key role in developing new therapeutic solutions. Studies in patients with major depression confirm the role of reactive oxygen species in depression’s pathomechanism. The mechanism of action is probably related to hyperfunction of the HPA-axis. Stress-induced increased blood cortisol levels accelerate glucose metabolism and ROS production. The group of ROS mediating the toxic effects of oxygen includes singlet oxygen free radicals and nitrogen free radicals: nitric oxide, nitrogen dioxide, as well as the non-radical forms such as: hydrogen peroxide, peroxynitrous acid, hypochlorous acid, and subthiocyanate [59,60,61,62,63]. Depression also results in reduced levels of antioxidants such as zinc, coenzyme Q10, vitamin E, and glutathione. This may contribute to an impaired defense against free radicals, leading to the development of oxidative and nitrosative stress (IO & NS) [64]. It has been shown that activation of the IO & NS pathway can lead to neuroinflammation and neurotoxicity. Increased ROS activity implies an increase in the activity of prooxidant enzymes, including xanthine oxidase (XO) and monoamine oxidase (MAO). MAO is an enzyme involved in the metabolism of serotonin.

Some studies on depressed patients have shown increased plasma NO levels, which can cause nitrosative stress and can lead to tissue damage [64,65]. Another pathway involved in the generation of oxidative stress is the nitrosative pathway. It can have detrimental effects on the structure of DNA, proteins, and fatty acids that make up the cell membrane [66,67]. One by-product of the oxidation of polyunsaturated fatty acids is malondialdehyde (MDA). MDA contributes to lipoxidation end products (ALE) creating, which as a consequence lead to weakening of the body antioxidant defense. Among a group of patients with diagnosed depression a substantial increasing in the concentration of MDA in serum has been observed [68,69]. In patients with depression both oxidative stress markers- F2-isoprostanes and 8-hydroxy-2′-deoxyguanosine (8-OHdG) turned out to be increased comparing with controls. The 8-OHdG is a derivative of deoxyguanosine and its concentration in cells indicates the formation of oxidative stress. The isoprostanes are formed in vivo in the process of peroxidation of arachidonic acid. Studies have shown that these compounds are indicators of lipid peroxidation. The relationship between depression and oxidative stress can be explained by the theory of allostasis and allostatic load. Long-term exposure to a stress response leads to the physical “wear and tear”. Furthermore, oxidative stress may lead to changes in the inflammatory pathway. People exposed to oxidative damage are at risk of developing somatic diseases due to high level of oxygen consumption of the brain [54].

Many studies confirm that a reduction in the number of nerve cells in the CNS can be observed in depressed patients. There are also increased levels of stress markers, including MDA, TNF-α, IL-1, and IL-6. Elevated levels of IL-1 may be associated with the development of depression, as confirmed by preclinical studies. Impaired social inhibition and reduced sexual activity were observed in test animals [70]. BDNF affects the formation of new dopaminergic, serotonergic, noradrenergic, and cholinergic connections. Due to BDNF’s ability to cross the blood–brain barrier (BBB), its blood concentration is thought to correlate with its concentration in the cerebral cortex. In a study performed on rats, significantly reduced plasma BDNF concentrations were observed in a group of depressed patients compared to a control sample [70,71]. It is possible that the use of antidepressants is associated with the elevated levels of BDNF in serum in patients suffering from depression. Oxidative stress-induced changes in the brain (especially in the hippocampus) may be central to the development of depression. Decreased hippocampal volume has been noted in depressed patients [72,73].

It is considered that the crucial factor in underlying the cause of AD is oxidative stress. Due to elevated oxygen consumption, the brain is particularly vulnerable to oxidative stress. Whether it is the cause of AD or its direct consequence has not yet been clarified [74,75]. It is associated with the presence of Aβ oligomers and is precisely about the inability to remove the neurotoxic molecule 4-hydroxynonenal (HNE). This dysfunction greatly contributes to the higher risk of disease progression [76]. Studies have shown that APOE is responsible for maintaining the body’s homeostasis by modulating the level of oxidative damage [77,78]. Inheritance of apolipoprotein E4 (APOE4) may be involved in the development of AD. This protein mutates and loses its ability to eliminate HNE [79,80]. Moreover, APOE4 can lead to the HNE formation and allows HNE to bind to the neuronal proteins [81]. The presence of the APOE4 gene causes failure of the neurons, possibly due to the absence of cysteine (Cys) residues, which plays a key role in preventing lipid peroxidation [82,83]. Hence, possession of the APOE4 allele was closely related to the high degree of the lipid peroxidation [84]. APOE4 can also induce oxidative stress indirectly, by interacting with mutated manganese superoxide dismutase (MnSOD) [81]. It has also been demonstrated, that people with the APOE4 gene have impaired glucose metabolism at an early age, before symptoms of the disease occur [81]. One hypothesis for AD is that the aging of the mitochondria is the major cause of the disease. This is because Aβ is located in the mitochondria where it can influence enzyme activity [85,86]. For this purpose, scientists have analyzed the activity of mitochondrial complexes (I, II, III, and IV). The greatest changes were observed for complex I and complex II. It was found that inhibition of complex I resulted in decreased production of tau protein and Aβ. Moreover, complex I is responsible for the association of oxidative stress with AD [86,87]. With the aging of the body, ROS accumulate, and thus the progression of oxidative stress. The enzymes contained in the mitochondria are essential for their detoxification. Mitochondrial dysfunction is most commonly seen in the areas of the brain involved in the AD formation [87,88]. Scientists have set out to investigate the relationship between the NADPH oxidase (NOX), responsible for the production of ROS and AD [89]. The NOX protein is, i.a., responsible for the induction of apoptosis and lipid peroxidation in cancer. It also mediates the production of ROS by activating the extracellular signal-regulated kinase pathway (ERK). When scientists examined the brains of AD mice, an increased concentration of NOX-responsive intermediates was observed. Several NOX inhibitor drugs are currently being studied as potential treatments for AD [6,90,91].

## 6. Antioxidants as the Prevention and Treatment of Depression and AD

Antioxidants are defined as endogenous and exogenous compounds, able to reduce or even prevent the destructive impact of free radicals on cells. In general, a radical is an atom, molecule, or ion that possesses unpaired electron on the valence shell [92]. Unpaired electrons make radicals highly reactive. If the balance between free radicals and antioxidants is not maintained, free radicals are able to initiate some destructive chemical reactions in human body. Thanks to their properties, antioxidants have anti-aging, anti-cancer, anti-cataract, anti-diabetic, anti-inflammatory, antibacterial, hepatoprotective, cardioprotective, and neuroprotective effects [93]. Antioxidants may be present in natural products and cells or be produced in a synthetic way. Endogenous antioxidants could involve enzymes like superoxide dismutase (SOD), glutathione peroxidase (GPx), and catalase (CAT), or work as non-enzymatic antioxidants, among which the most popular are: melatonin, glutathione, uric or lipolic acid, and bilirubin [94]. However, carotenoids, vitamin E, C, A, natural flavonoids, or other compounds are included as exogenous antioxidants [93]. It is also worth mentioning that synthetic antioxidants, despite being mainly used to prevent food spoilage, have also significant impact on human enzymes and DNA [95]. The protection of antioxidants to biological system is based on three main ways:

- Inhibition of the generation of new radicals (action of SOD, CAT, Se, Cu, Zn);
- Catching free radicals to prevent chain reactions (carotenoids, vitamins E, C);
- Vanish any disturbances caused by free radicals (lipases, proteases) [96].

Currently, effective curing of the nervous system diseases constitutes one of the greatest therapeutic challenges. It is not surprising that many substances with antioxidant properties have been investigated as a potential weapon to overcome nervous system disorders. Specifically, scientists are trying to find an effective way to prevent and cure depression and AD, which are considered some of the most common nervous system disorders of the XXI century.

### 6.1. Antioxidants and Depression

According to World Health Organization (WHO), depression seems to be the most common disorder of the nervous system of our times [97]. Despite the availability of many anti-depressants able to treat this neuropsychiatric dysfunction, scientists are still looking for new substances that would be both effective and without severe side effects [98]. There is no doubt that one of the reasons of the occurrence of depression is oxidative stress. The increasing level of ROS, which is caused the disturbance in the prooxidant–antioxidant balance (PAB) and entails peroxidation of lipids [99]. Therefore, one of the groups with potential application in treatment and prevention of the disclosure of depression seem to be antioxidants. From analyzing many studies and clinical trials, we find that the most promising results are antioxidants such as: curcumin, zinc, selenium, vitamin E and saffron.

#### 6.1.1. Natural Substances

Many antioxidants with medicinal properties are acquired from plants. One of them is quercetin. It is a compound flavonoid, which arises as a secondary metabolite [100], able to activate some antioxidant enzymes and decrease lipid peroxidation [101,102]. Quercetin is present in our daily diet mainly in some fruits, vegetables, tea, and wine [103]. Noticing its antioxidant activity, Samad et al. (2018) have analyzed the effect of quercetin to memory function, reduction anxiety, and depression [100]. During the clinical trial, animals were being repeatedly administered with this compound, whose impact on stress responses, cognitive function, cholinergic and serotonin reaction was then observed. Exposure of the mice to stress caused neurochemical changes, which allowed scientists to obtain proper animal models of depression [104,105]. The results have clearly shown that administration of quercetin reversed symptoms of depression and anxiety in stress animal models [101]. Flavonoid was capable of inhibiting activity of MAO that prevents organism from the excessive amount of free radicals and oxidative stress [106]. It also caused decreasing level of 5-HT, which improves pro-cognitive and antidepressant efficiency. It is certain that these promising results of clinical trial were partially the effect of strong antioxidant properties of quercetin.

The *Conyza canadensis* medical properties have been known for a long time [107,108,109]. The most potent compounds have been identified in oils isolated from various parts of plant. El-Akhal et al. tried to discovered the connection between antioxidant and antidepressive activities of that plant [110]. For that reason, they obtained the aqueous extract from aerial parts of *Conyza* and administered it to scopolamine (Sco) rat models. During the tests it was found that the extracts are rich in catechins and flavonoids. The antidepressant and anti-anxiolytic impact of extracts were investigated by the Elevated Plus-Maze (EPM) and The Forced Swimming Test (FST). The Sco rat model proved the potential antidepressant activity of aqueous extracts from aerial parts of *Conyza canadensis*, which could have potential application in future therapy of depression. The extracts obtained from: *Valeriana officinalis*, *Centella asiatica*, *Acanthopanax senticosus*, *Houttuynia cordata*, *Withania somnifera*, *Dan Zhi*, *Campsis grandiflora*, *Psoralea corylifolia*, and *Corydalis yanhusuo* have specified to more than 15 Trolox (water-soluble analogue of vitamin E) equivalents per mg extract that have showed antioxidant activity. The mentioned extracts had the ability to increase the resistance to heat and osmotic stress, extending lifespan of the nematodes and also significantly decreasing the level of ROS in mutants. Moreover, the extracts from tested plants in the nematode model have shown very promising results in increasing its resistance to stress. However, further analyses are needed, so as to unravel underlying specific mechanisms [111]. Walnuts from *Juglans regia* have a high content of flavonoids, proanthocyanidins, phenolic acids, melatonin, folate, vitamin E, selenium, and juglone. These chemical compounds are antioxidants. Moreover, walnuts contain compounds with highly anti-inflammatory properties, such as plants’ omega-3 fatty acid and n-3 α-linolenic acid (ALA). All of these substances have a neuroprotective effect on cerebral tissue [112]. Walnut extract has positive effect on the struggle with oxidative stress induced by Aβ. The walnuts components such as melatonin, flavonoids, γ-tocopherol, and ellagic acid can neutralize the excess of ROS. High consumption of large amounts of nuts increases total antioxidant capacity and reduces plasma lipid peroxidation [113]. The researchers have reported considerably less frequent depressive symptoms in people who included walnuts in their daily diet, compared to a control group who did not. The risk of depression and stroke was meaningfully lower among people who eat walnuts in a nut-enriched Mediterranean diet compared to the control group (people who used a low-fat diet) [112].

#### 6.1.2. Micro- and Macroelements

Zinc (Zn) is considered to one of the most significant microelements that affects the nervous system. So far, many analyses and studies investigating the relationship between Zn and depression have been carried out [114,115,116]. Undoubtedly, as confirmed by some researchers, regular and appropriate dosage of Zn reduces the symptoms and disorders caused by depression [117]. Interestingly, the improvement of patients’ health due to administration Zn was also observed in the absence of anti-depression drugs. The highest level of Zn intake during a study was correlated with the highest (about 28%) reduction of the risk of depression [117]. It is also suggested that Zn has the most effective and mild approach in terms of moderating depression. The capacity of Zn to prevent or even reduce the regression of depression may result from its antioxidant properties [118,119]. It has been reported that suitable dosage of Zn is able to decrease the level of C-reactive protein (CRP), whose excessive level in human cells is observed due to depression [120,121]. Zinc is also able to prevent cells from the adverse results of lipid peroxidation, which is meaningful in MDD treatment [122].

The connection between selenium (Se) and depression has not been discovered as well as the associations between zinc with depression [119]. However, some of the latest reviews have demonstrated strong correlation between the appropriate level of Se and the symptoms of depression [123,124]. Interestingly, both too much and too small a quantity of this microelement caused dysregulation in neuronal areas as a result of occurrence oxidative stress and inflammation. The scarcity of selenium has been associated with abnormal production of pro-inflammatory cytokines, such as IL-6, CRP, and growth differentiation factor-5 (GDF-5) [125]. The availability of this micronutrient is also indispensable for some enzymatic reactions because among many antioxidant enzymes some of them like GPx, selenoprotein P, and thioredoxin reductases are selenoproteins. They protect cells not only from the effect of damaging ROS, but also inhibit lipoperoxidation provided longer life of undamaged cells [126]. On the other hand, the metabolites which have arisen due to excessive amount of selenium are probably pro-oxidative [127]. Wherefore, defining effective dosage of selenium (not too small and also not too high) seems to be the greatest challenge for present scientists looking for a new way to treat or prevent depression [119].

Magnesium (Mg) is one of the most important elements when it comes to proper action of numerous enzymes and processes, mainly in the nervous system [119,128]. Deficiency of magnesium could be one of the risk factors of the mental disorders [129]. Moreover, some researchers have found that decreasing the level of Mg could lead to depression [130], as confirmed both in animal and human studies [131,132]. It has been suggested that supplementation of Mg might be one of the methods to prevent and treat depression. Although the mechanism which underlies the association between level of Mg and depression has not yet been discovered, it is suggested that one of them is connected with antioxidant and anti-inflammatory processes [119]. Previous studies have pointed out the correlation between the low level of magnesium and the higher level of inflammatory factors CRP, Il-6, or TNF- α [133,134] are responsible for destructive processes during depression. These assumptions have considered the new perspective of pathogenesis and treatment the depression; therefore, it is important to gather more evidence from clinical trials.

### 6.2. Antioxidants and AD

AD is multifactorial disorder with not- fully known etiology; hence, new methods of treatment are still being sought. At present, the treatment options of AD are very impecunious. The specialists usually prescribe medications which accomplish memory and concentration without changing the progression of AD dementia. The drugs reduce only the result of AD, without affecting its cause. Moreover, not only treatment but also prevention of disease outbreak is very important. In the recent years, some clinical trials and experiments have shown that antioxidants, due to their numerous properties, could relieve patients with AD. Among countless compounds with antioxidants properties, the best results have accomplished: vitamin E, coenzyme Q10 melatonin, polyphenols, curcumin, and selenium. The main mechanism of action of these substances is based on protecting the cells from harmful amount both extra- and intracellular ROS and H_2_O_2_ [135].

#### 6.2.1. Natural Substances

Some plants, thanks to their activity as inhibitors of acetylcholinesterase (IAChE), could be used in the treatment of AD. One of them is *Hedychium gardenarium*. Arruda et al. have isolated the essential oils from the leaves of *Hedychium gardenarium* and tried to use them in aromatherapy as co-adjuvant during treatment of some cognitive disorders, e.g., AD [136]. It was observed that these essentials oils have had acted both as antioxidants and IAChE, which could be really effective in sufficient therapy of AD. The increasing level of acetylcholinesterase (AChE) in cells contributes to prevention of damage caused by free radicals. Additionally, these volatile compounds used in aromatherapy are lipophilic and small, thus they could cross the BBB and avoid the secondary effect. Brimson et al. in 2012 [137] have received both ethanol and hexane extract from the root and leaf of *Rhinacanthus nastus* so as to identify and compare their capacity to protect neuronal cells from death. During all studies that were carried out on hypoxia model [138], it was proved that the achieved extracts contain various flavonoids, phenolic compounds, sterols like stigmasterol or β-sitosterol and triterpenoidlupeol. All these compounds were responsible for advantageous impact on neurons, mainly prevention of toxicity of Aβ and glutamate on HT-22 cells (cultured mouse clonal hippocampal cells). The ethanol extracts were proven to exhibit the most potent antioxidant activity. Although the mechanism of action of the combination of all these compounds has not been discovered yet, there is no doubt that thanks to the strongest capability to scavenging free radical the ethanol extracts of *Rhinacanthus nastus* could be perceived as a potential neuroprotective agent in treatment of cognitive disorders. Another plant with proved activity as IAChE is *Gossypium herbaceam*. Scientists from China have obtained the extract from its flowers and identified some flavonoids, which have possessed antioxidant properties [139,140]. So far, the specific compound (flavonoid) responsible for capability of flower’s extract to decrease the level of free radicals has not been identified. However, using the rat models of AD it has been observed a beneficial association between the activity of the extract as antioxidant and IAChE activity, which could be helpful in designing the new way of treatment AD. Listing some species of plants with confirmed activity as IAChE it also is also worth mentioning some species of the *Lamiaceae* family. Vladimir-Knežević et al. have examined a wide group of ethanol extracts obtained from many species of the *Lamiaceae* family [141]. During the tests the strongest activity (over 75%) against AChE was noticed from the ethanolic extracts of *Salvia officinalis*, *Teucrium arduini*, *Teucrium chamaedrys*, *Teucrium montanum*, *Teucrium pom*, *Satureja montana*, *Mentha x piperita*, *Mentha longifolia*, and *Thymus vulgaris*. The factor responsible for this activity in almost all the mentioned species (except of *Teurcium species*) was rosmarinic acid. Additionally, all 26 analyzed extracts had antioxidant activity. The co-operation of these two significant activities meant that some species of *Lamiaceae* family, due to their content of many phenolic and terpenic compounds, could be used in prevention and treatment of many diseases, including cognitive disorders like AD. Another species belonging to this family is an endemic Bolivian plant called *Azorella glabra*; its medicinal properties have been used since centuries to treat many diseases [142]. However, a possible use of the plant to prevent and treat neurodegenerative disorders like AD was mentioned for the first time in 2019 by Faraone et al. [143]. The scientists have investigated its aerial parts. The presence of some terpenes in ethanolic macerate is probably responsible for their antioxidant behavior and they inhibit cholinesterase AChE and butylocholinesterase (BuChE) activities. The analysis of the results of all performed tests allowed the scientists to conclude that co-operation of these activities may be the key to success in effective treatment of disorders, such as like AD caused by oxidative stress. *Centella asiatica* is a multifunctional plant that has been used for a long time in traditional Chinese and Ayurvedic medicine. So far, numerous studies in rodents and humans have proved that the plant possesses pro-cognitive [144,145,146], neuroprotective [147], or antioxidant [148] properties mainly due to the high content of pentacyclic triterpenoid saponins like asiaticoside, madecassoside, and their sapogenins [149]. In 2020 Zulikha Hafiz et al. decided to verify whether the extract from *Centella asiatica* is capable of reducing oxidative stress and inhibit the activity of AChE both in vitro and in vivo models [150]. Having carried out the tests, the scientists achieved very promising results that may be genuinely useful in terms of developing new potential methods of treatment of AD with the use of that medicinal plant. In 2019 Matthews et al. have focused on the ability of *Centella asiatica* to improve the memory and induce better antioxidative response [151]. The scientists aimed at discovering the mechanism of action underlying better memory. They conducted in vivo studies with both male and female mice models AD (5XFAD). During the experiments, a significant improvement in memory of mice regardless of their sex was observed. Moreover, the extract turned out to decrease the level of SOD1 (one of the markers of oxidative stress in cells) connected with Aβ plaque in mice cortex and hippocampus. These effects were probably caused by multiple interactions between antioxidants and pro-cognitive components (specifically triterpenes and caffeoylquinic acids) of water extract from *Centella asiatica* (CAW). Medicinal properties like anti-inflammatory, antibacterial, antioxidant, of *Grewia species* have been used in traditional treatment for a long time [152]. Many active compounds have been isolated from some *Grewia spp*. In 2020 Bari et al. were able to isolate and investigate antidiabetic, antioxidant, and anticholinesterase activity of four new compounds [153]. The best results were obtained for 2,2′-(1,4-phenylene) bis (3-methylbutanoic acid), which was labelled XII in that experiment. The strong potential of this compound to inhibit not only AChE but also BuChE was observed, which may allow researchers to better design efficient treatment of AD. There is growing evidence that inhibition of these two types of AChE may provide better results in both the prevention and treatment of AD. Furthermore, capacity of that new compound to scavenge free radicals ensures co-operation of all these activities and may be a potential way of treatment for AD. In *Ziziphus mucronata* leaves the extract of five cyclopeptide alkaloids (sanjoinines- A, B, F, G1, and G2) were identified [154]. For that reason, in 2019 Foyet et al. have investigated the potential anticholinesterase and antioxidant activity of these compounds [155]. In order to do so, they obtained hydromethanolic extract from its leaves and administered it to mice in (Sco)- induced model of dementia. The impact on mouse memory was observed during Y-maze, radial maze, and novel object recognition tests. Indisputably, the improvement of cognitive processes (including memory) was due to the reduction of oxidative stress. The level of CAT and SOD in mice brains increased, whereas the levels of MDA decreased in comparison with the control group. Moreover, the amount of AChE significantly raised that suggests the un-doubtful connection between cholinergic endogenous system and oxidative stress.

Interestingly, the plant kingdom is not the only kingdom rich with compounds showing activity as IAChE. *Morchella esculenta* is a mushroom which contains polysaccharides, mainly β-glucans with many properties. During a study in 2021 Badshah et al. [156] have decided to define the association between free radical scavenging and AChE activity of two forms of these polysaccharides, proteinized and deproteinized. Their capability to inhibit AChE was compared with galanthamine as a reference standard. Better blocking of enzyme was noticed in the proteinized polysaccharides of *Morchella esculenta*. Their inhibitory potency was not as good as that of galanthamine, which allowed us to conclude that using of these proteinized polysaccharides may be useful in polytherapy with other drugs so as to combat neurodegenerative disorders like AD. Moreover, the regular intake of this mushroom improves the memory and cognitive function of older people, which is another advantageous effect.

*Ferula assafoetida* is popular spice in Indian and Nepali cuisine. Moreover, medicinal properties of this herb are known and used for centuries in a traditional medicine. The secretion of the plant’s rhizome, tap roots, and gum-resin is known as “Asafoetida”, “Anghouzeh”, “Khorakoma”, and “Anguzakoma” in Iran. The ole-gum-resin of *F. asafoetida* has antioxidant, anti-diabetic, and antihypertensive effects. In rat models, researchers have proved that higher doses of extract (400 mg) improve memory of animals. It was reported that *Ferula assafoetida* has AChE in vivo inhibitory property in snail nervous system. Probably, the memory improvement on rats can be linked to the inhibitory effect on AChE in the rats’ brains [157,158]. *Nigella sativa* seeds are a well-known Persian food spice. Chemical components of seeds are: carbohydrate, oil, fiber. and protein. The oil contains acids such as: linoleic, oleic, palmitic, arachidic, myristic, stearic, and eicosadienoic. The main *N. sativa* phenolic compounds obtained from seeds are p-cymene (37.3%), thymoquinone (TQ) (13.7%), carvacrol (11.77%), and thymol (0.33%) [158]. Antioxidant effects of *N. sativa* in the rats’ brains are improved Sco-inducted learning and memory with reduced AChE activity and oxidative stress [158]. *N. sativa* and TQ reduce IL-10, MDA, and NO level in human serum, and both reduce oxidative stress. In a clinical trial, 500 mg of *N. sativa*, when compared to the placebo group, improved the cognition function, memory, and attention. This dose also caused mood-stabilization and antianxiety properties in the human model [159]. The main components of *Zataria multiflora* oil (which has documented antioxidant properties in vitro) are carvacrol, thymol, γ –terpinene, β-caryophyllene, and p-cymene [158]. Moreover, in a rat model, Aβ-caused by neurocognitive weakening could be restored by administration of *Zataria multiflora* essential oil [158]. *Withania somnifera* is called an Indian ginseng, winter cherry, or ashwaganda. The main constituents of mentioned plant are: anferine, isopellertierine, sitoindoside (VII, VIII) withanolides, withanoloides, and withaferins. Other compounds which could be found are: somniferine, somniferinine, somnine, withanine, pseudo-withanine, withananine, cuscohygrine, tropine, pseudo-tropine, choline, and 3-a-gloyloxytropane [160,161,162,163]. *Withania somnifera* has many biological properties such as antioxidant, anti-inflammatory, neuroprotective, anti-depression, anti-stress, and immunomodulatory [164]. In a double-blind, randomized, and placebo-controlled trial is has been proven that the administration of ashwaganda-root extract (600 mg/day for eight weeks) increases memory, executive function, and information processing speed and also enhances cognitive functions in adult patients suffering from a mild mental weakening [165,166]. The extract with withaferin A and sitoindoside VII-X (50 mg/kg p.o. for two weeks) reversed the cognitive deficit caused by ibotenic acid and reduced cholinergic markers (e.g., acetylcholine- ACh and choline acetyl transferase- ChAT) in rats [162]. Sitoindosides VII-X and zaferin administrated variably (40 mg/kg for one week) advantageously changed the activity of AChE and increased M1- and M2-binding to muscarinic receptors in some regions of brain [162]. Both Withanolide A and Withaferin A have probably strong immunomodulatory effect in microglial cells (BV-2) by activating the pathway of Nrf2 which leads to the formation the proteins with neuroprotective properties [164]. *Thymus vulgaris* is an herb used as a spasmolytic, antioxidant, and anti-inflammatory traditional medicine. Essential oil of *Thymus vulgaris* (TEO) improves cognitive function and memory by acringon cholinergic neurons of Sco-induced *Danio rerio* (zebrafish) in a model of memory impairments. Different doses (TEO 25, 150, 300 µL/L) were administered once a day (for 13 days) to zebrafish. Additionally, 30 min before behavioral tests the memory impairment in animals was induced by 100 μM of Sco. The results indicated that TEO enhanced Sco-induced increasing of AChE activity which determined it as a potential therapeutic and/or preventive substance in the management the memory disturbance in animal models. However, more research and clinical trials are needed in order to prove effectiveness of the essential oil of *T. vulgaris* in prevention of AD [167]. Ferulic acid is a compound commonly found in the plant world. Products containing ferulic acid include fruits, vegetables, coffee beans, and cereal bran. It is estimated that the daily intake of ferulic acid in people whose diet is rich in these products is around 500–1000 mg. It presents anti-inflammatory and antioxidant properties, due to the prevention of senile plaques associated with the accumulation of Aβ in the brain [168].

#### 6.2.2. Pigments

Lycopene is a carotenoid obtained from plants such as watermelons, tomatoes, or papayas [169]. The proper supplementation of lycopene can improve cognitive function in Tau transgenic mice [170,171]. Moreover, 10 weeks of lycopene treatment has shown to attenuate mental impairment in streptozotocin-inducted rats. There occurred a decrease of AChE activity and an increase in enzymatic activities of CAT and SOD with simultaneous decreased levels of NO in the cerebral cortex and hippocampus [172]. Lycopene, as an antioxidant, is tested for antidepressant effects. Trials have shown that 3 days pretreatment with lycopene (10 mg/kg) reverses lipopolysaccharide-inducted increase in plasma pro-inflammatory cytokines. It is correlated with the neuroinflammation hypothesis of depression, yet verification of this hypothesis requires further studies.

β-carotene is an organic red colored compound, present in fungi, plants, and fruits. It is one of the carotenes–isoprenoids synthesized biochemically. The antioxidant properties of this pigment were proved during a study on mice, assessing the effectiveness of β-carotene antioxidant strength mechanisms against intracerebroventricular injected streptozotocin-induced memory impairment in mice. The passive avoidance, EPM and open field paradigms were used to define the cognitive performance of animals [173]. AChE, Aβ, GPx, SOD, and CAT were analyzed post-mortem in the cerebral tissue. The experiments showed that administration of β-carotene in streptozotocin-induced cognitive deficit mice which resulted in inhibition of AChE activity and antioxidative effects.

Anthocyanins are natural antioxidants and a subgroup of flavonoids. They are present in blueberries, violet grapes, black currants, and red wine [174]. Anthocyanins are responsible for the characteristic color of these products, from blue to red. These groups of phytotherapeutics are commonly used in folk medicine [175]. Anthocyanins can be divided into internal (indirect) or external (direct) antioxidants. The direct effect of free radical scavenging is due to their potential to donate electrons/hydrogen in their structure. Anthocyanins have a high radical oxygen absorption capacity (ORAC), which may in part account for a neuroprotective effect. Similarly, in in vitro models with H_2_O_2_ damage and Aβ peptide-induced toxicity, anthocyanins directly clear intracellular ROS formation [176]. Indirect antioxidant activity of anthocyanins could be a result of restoration and enhancement of endogenous antioxidant enzyme activities- SOD, GPx, and CAT. All neuroprotective effects (also based on arising glutathione concentration and decreasing glutamate induced neurotoxicity) are the reason of growing popularity of anthocyanins in treatment neurodegenerative disorders [176].

#### 6.2.3. Melatonin

Today it is known that most living organisms, both animal and plant, synthesize melatonin. In spite of being synthesized in the pineal gland and acting as a hormone, it is increasingly reported that melatonin is involved in many other activities, including as an antioxidant [135,177]. The older we are, the less melatonin is produced in our bodies. For that reason, it is suggested that the level of melatonin may be one of the factors of degenerative diseases connected with age [135,178,179]. On the other hand, it was proved that this compound has twice higher capacity to eliminate ROS from human body than vitamin E [180]. On the basis of this observation, in 2013 He et al. have investigated how the melatonin agonist Neu-P11 (which had been used in treatment of insomnia) could impact memory of rat AD model [181]. The results of clinical trials were very promising. It has been demonstrated that Neu-P11, like melatonin, improves the animals’ memory and may thus constitute a new compound for treatment for the AD effects. During other clinical trials, the rats were administered with melatonin per os [182]. It was noticed that the production of pro-inflammatory cytokines was inhibited. Therefore, it was proved that melatonin both decrease the level and reduces the toxicity of Aβ, which is the reason of neurodegeneration in patient’s brain [183]. Summarizing, the use of melatonin can possibly be a potential way for treatment of the AD.

#### 6.2.4. Coenzyme Q10

Coenzyme Q10 (CoQ10) is classified to the group of lipid-soluble benzoquinones, which demonstrate antioxidant properties [184]. Currently, this compound is investigated as a neuroprotective substance that can prevent cells from a complicated death process and restore their neuronal activity at the same time [185]. These properties may suggest the potential use of CoQ10 as an antioxidant in treatment and possibly in prevention of neuronal diseases, especially AD. In 2019 Komaki et al. investigated the efficiency of CoQ10 in rat model both before and after induction of AD [186]. Their findings have shown that supplementation of CoQ10 increased the long-term potentiation (LTP) of hippocampus both in group of rats with AD (induced by administered Aβ) like in the group of healthy rats. Moreover, CoQ10 caused promising changes to the oxidant/antioxidant balance. Scientists have also confirmed the previous findings, i.e., that CoQ10 is able to meaningly reduce the activity of antioxidant enzymes such as: SOD, CAT, and GPx and decrease the ROS accumulation in various structure of the brain [187,188].

Attia and al., in 2020, also tried to prove the efficiency of CoQ10 as a prevention in neuronal disorders with and without collaboration with biotin [189]. In order to do so, they administered rat models of AD single CoQ10 and CoQ10 in addition with biotin. The best results gained the combination of both of these substances. However, also the single biotin showed the capacity to reduce the level of oxidative stress and excessive accumulation of Aβ.

#### 6.2.5. Carnosine

Carnosine is a natural occurring dipeptide. This compound found is in tissues and organs such as spleen, kidneys, and in the brain, in small amounts. It appears that in patients with probable AD the plasma carnosine levels are significantly lower than in healthy individuals. Carnosine deficiency is synonymous with reduced cognitive function. Carnosine may act as a neurotransmitter, a compound that enhances the immune system function and a modulator of nitric oxide metabolism. It enhances cell metabolism and acts as an anti-ageing agent. Human studies have been conducted using carnosine and anserine, which is a methylated analogue of carnosine. The results have shown significant improvements in cognitive function, memory, and physical activity. Thus, it can be concluded that the exposure to carnosine deficiency contributes to cognitive impairment and thus to the development of dementia and AD [190].

#### 6.2.6. Vitamin E

One of the fat-soluble vitamins with significant antioxidant properties is vitamin E—a group of eight compounds with similar structure and solubility in lipids, tocopherols and tocotrienols. Each class has four homologs α (alpha), β (beta), γ (gamma), and δ (delta), depending on the number and localization of methyl groups [191]. The most significant, common and most bioavailable form of vitamin E is α-tocopherol [192,193]. Vitamin E is a multifunctional nutrient that maintains the integrity of cell membranes. It is also crucial for proper development of human tissues, especially the brain and nervous system [194]. A daily recommended intake of vitamin E in Poland is 6 mg for kids, 10 mg for men, 8 mg for women, and 20–50 mg for older people (above 75 years old) [195]. The beneficial and important impact of vitamin E on the nervous system has been known since the beginning of the XX century, when Evans and Burr observed and then described the offspring of rats maturing without vitamin E in their diet [196]. In the following years, more and more clinical trials have shown that the level of vitamin E in organism affects the occurrence or lack of disorders connected with the nervous system [197,198].

Vitamin E exhibits the strongest antioxidant properties resulting in its capacity to eliminate excessive quantity of free radicals in human cells and stop peroxidation of lipids. All these neuroprotective processes prevent cells from hemolysis and their premature death [199]. The transport of vitamin E across neuronal system depends on availability of a specific transporter called α-tocopherol transfer protein (α-TTP) [200,201]. The results of many studies have shown that scarcity of this carrier is observed in patients suffering from AD [201]. Therefore, is has been proven that decreased levels of vitamin E in plasma cause neuronal disorders and are indispensable for proper activity of neuronal cells [201]. It should be mentioned that vitamin C, one of the most important natural antioxidants, is often said to be a supporter of vitamin E due to protection of lipoperoxidation [93]. Vitamin C, being a water-soluble electron acceptor, prevents the membrane from excessive accumulation and lipid-damaging of oxyradical (unpaired electron), which are side-products of non-balance dietary with vitamin E [202]. In 2002 Morris et al. proved that simultaneous intake of both vitamin C and E could possibly decrease the risk of the AD occurrence, yet it should be confirmed by further research [203]. The scientists have also tried to define whether dietary intake of only vitamin E prevents AD. Actually, better results were found for patients who had higher consumption of vitamin E with food. However, these quantities of tocopherol have not delayed the progression of dementia [204]. Furthermore, a daily dosage of vitamin E should be determined and followed individually for each patient, since in 2005 Miller et al. proved excessive intake mortality, as a result of surplus in the plasma redox potential [202,205].

Aβ is considered as a main factor of AD disorders caused by ROS. Due to some clinical trials with animal models, it has been proven that vitamin E improves their memory and cognitive properties [206,207]. Moreover, it was observed that the longer supplementation of vitamin E the better improvement of efficiency of disorders treatment [208]. In another clinical trial, the mice were administered α-tocopherol in different dosages [209]. The scientists observed a significantly decreasing level of insoluble tau proteins which excessive accumulation is observed in patients with AD. Despite supplementing two various dosages of vitamin E, no significant differences between them were observed. Therefore, the study suggests that influence of vitamin E is not depended of dosage. It is worth remembering that the complex of vitamin E also includes tocotrienols. More and more studies reveal that they probably have better and stronger activity than tocopherols antioxidant, which may constitute another effective way of AD treatment [210,211].

#### 6.2.7. Microelements

Selenium is one of the microelements that have proven antioxidant properties. It affects the cells (mainly in the nervous system) through selenoproteins, such as GPx, thioredoxin reductase, selenoprotein P, selenoprotein R, and selenoprotein M [135,212]. Recently, it was noticed that the decreasing level of selenium is connected with age [213]. Moreover, according to clinical trials with two groups of patients—with and without AD—it was found that the patients with that disorder have significantly lower levels of selenium than healthy people of the same age [214]. This observation allows us to assume that the intake of proper quantity of selenium may slow down cognitive disorders associated with AD [215]. In order to prove this assumption Ishrat et al. administered rat model of AD sodium selenite [216]. Results of this trial have indicated that sodium selenite is able to prevent both oxidative damage and cognitive disorders. Another trial with the use of sodium selenite in AD mice models proved that this substance significantly activates protein phosphatase 2A that prevents neurodegeneration, excessive tau pathology, and other neuronal functional disorders [217]. These results may prove that selenium could be used as a supportive compound in patients suffering from AD.

#### 6.2.8. Chemical Compounds

Astaxanthin (3,3′-dihydroxy-β,β′-carotene-4,4′-dione) is an orange–red pigment that belongs to xanthophylls. It is a lipophilic carotenoid, whose structure contains a polyene chain and two terminal hydroxyl groups, which determine its low solubility in water. It is found in microalgae such as *Haematococcus pluvialis*, but also in salmon and shrimps [218]. Astaxanthin, due to the presence of the polyene chain in their structure are able to trap radicals, which indicates strong antioxidant properties. Many studies have shown that astaxanthin’s effects are more potent than α-tocopherol, and about 10 times more potent than other carotenoids like: β-carotene, lutein, and zeaxanthin. Astaxanthin may also be important in the prevention of cardiac disease. It also has anti-aging and neuroprotective effects [218,219] and has the ability to reduce ischemic brain injury and may be used in pretreatment for stroke [220]. The antioxidant and anti-inflammatory activity combined with the ability to pass through the BBB makes astaxanthin a promising substance that may be applied in neurodegenerative diseases. Several previous studies clearly indicate that astaxanthin is able to form hydrogen bonds and van der Waals interactions with Aβ and prevent its accumulation and protect hippocampal cells from the deleterious effects of Aβ [219,221]. The unsaturated bonds in its structure limit its therapeutic use due to its instability and susceptibility to oxidation and photooxidation reactions. Administration of astaxanthin in stealth solid lipid nanoparticles (AST-SSNLs) favorably affects the pharmacokinetic and pharmacodynamic parameters of the substance. SNLs are colloidal transporters composed of solid biodegradable lipid (GRAS). Surface stabilization with surfactant (polysorbate 80) helps to avoid opsonization by immune cells. The diameter of the nanoparticles was <200 nm for parenteral administration [219].

Methyl gallate is a compound found in *Terminalia chebula* and *Terminalia arjuna*, among others. It exhibits antioxidant properties, inhibition of AChE as well as BuChE, blocks Aβ aggregation and destabilizes mature fibrils [222]. However, its poor stability, solubility, and inability to pass through the BBB greatly hinder its use in AD therapy [223].

Starch is a widely distributed polysaccharide. It is composed of two biopolymers amylase and amylopectin. Depending on its origin, starch can vary in its properties [224]. The medium porosity and small fractures in the nanoparticle structure allow the active ingredient to be transported to the outer layers [225]. Starch-encapsulated methyl gallate (SEMG) exhibited the previously mentioned activities in addition to less severe side effects [223].

### 6.3. Antioxidants and Both Depression and AD

#### 6.3.1. Natural Compounds

The pro-health properties of curcumin have been known for many centuries. The beneficial impact on maintaining proper homeostasis is a result of anti-oxidant and anti-inflammatory effect of this popular spice [226]. It is considered that the presence of the hydroxyl and methoxy groups in the structure of curcumin gives these advantageous properties [227]. The main use of curcumin is described in terms of trials testing new possibilities of treatment of mental disorders including depression, both in animal models and humans [228]. Interestingly, many preclinical trials have shown the efficiency of curcumin similar with antidepressant synthetic drugs like fluoxetine and imipramine [229]. However, the most effective mechanism of action is based on the antioxidant mechanisms [230]. Curcumin has the ability to inhibit both MAO-A and MAO-B [231], which means that it is able to prevent the accelerated decompositions of neurotransmitters involved in proper functioning of the neuronal system. Franco-Robles et al., during a large clinical trial, have noticed another advantage of curcumin, a capability to restore the level of BDNF, which may consist of a therapeutic potential way to repair oxidative damage indexes [232]. The ability to inhibit protein kinases (PK) and the mitogen-activated protein kinase (MAPK) pathway is also responsible for the anti-inflammatory effect of the curcumin. This leads to inhibition of the synthesis of pro-inflammatory cytokines and decreases the level of CRP which is an inflammation marker. Curcumin inhibits lipid peroxidation and neurodegeneration of neurons are caused by oxidative stress. Preclinical studies by Qian et al. showed potential antioxidant activity [233]. The method was based on measuring the concentration of the lactate dehydrogenase (LDH) and MDA. Curcumin lowered the LDH and MDA levels [233,234].The effect of curcumin on the development of AD disease has been studied in in vivo and in vitro models. Curcumin significantly reduced the neurodegeneration process in the hippocampus. Its administration also contributed to the inhibition of APP β cleavage and deposition of Aβ deposits in the hippocampus compared to the ad group [235]. Even though it is proven that curcumin has strong free-radical-scavenging activity [236], better results are provided by the use of curcumin structural analogues [237,238]. Recently, scientists were able to synthesize a new compound with potential use in the treatment of AD. It is a new IAChE, which is a combination of galantamine and curcumin. In ex vivo tests, the fusion of the two compounds is more effective than their separate administration [239]. This combination is based on the strong antioxidant properties of curcumin and the AChE inhibitory activity of galantamine [240]. In addition, in vivo tests carried out after oral administration of the compound to mice showed low toxicity (LD_50_= 49 mg/kg) [241]. Curcumin has also shown to have a positive effect on anxiety symptoms that often accompany depression. This may be due to inhibition of MAO that breaks down serotonin and the conversion of α-linoleic acid to docosaesaenoic acid (DHA), which has an anxiolytic effect [228,242].

Saffron is a plant material obtained from *Crocus sativus*. It is a substance rich in carotenoids (crocin, crocetin), monoterpene aldehydes, and flavonoids. Research has shown that saffron affects the functioning of the nervous system. Therefore, it was decided to investigate the potential use of saffron in the treatment of depression in preclinical studies [158]. Rodent studies have shown a significant relationship between saffron intake and development of depression. The following effects of saffron may be important in depression:

- Antioxidant effect related to lowering the level of lipid peroxidation markers;
- Anti-inflammatory effect by inhibiting the activity of cyclooxygenase-1 (COX-1), cyclooxygenase-2 (COX-2), and the production of prostaglandins;
- Hypothalamus–pituitary–adrenal-modelling effects, which may result from a reduction in the sensitivity of the HPA axis to stress;
- Serotoninergic effects resulting from antagonism of 5-HT2C receptors;
- Neuroprotective effects—crocin, which is a component of saffron, protects the neurons of the cerebral cortex and hippocampus against the effects of oxidative stress [158,243].

Most likely, this is due to the activation of dopaminergic, serotonergic, and noradrenergic systems, associated with the pathogenesis of depression. The carotenoids of saffron are mainly responsible for this action [244]. The relation between depression and other neurodegenerative diseases, such as AD, is also important. The protective effect of crocin and crocetin on neurons has been confirmed in several clinical studies [245]. It results from the reduction of oxidative stress and inhibition of the Aβ aggregation. Reported studies have shown that administration of *Crocus sativus* and saffron extract to patients with mild-to-moderate AD improved the cognitive functions as effectively as donepezil. The side effects of both donepezil and saffron were comparable. The main difference was that donepezil triggered vomiting and the saffron extract did not. Moreover, the saffron extract administration in a dose 30 mg/day for six weeks shows similar effects as fluoxetine and imipramine (100 mg/day) in the treatment of depressive disorders [158]. A double-blind clinical trial proved that co-administration of hydro-alcoholic extract of *Crocus sativus* (80 mg) and fluoxetine (30 mg/day) was effective to treat mild-to-moderate depression [158].

In traditional medicine, *Rosmarinus officinalis* has been used as an antispasmolytic, mild analgesic, and for treatment of depressive episodes and emotional upsets [246]. Rosemary exhibits anti-inflammatory, antioxidant, anti-apoptotic, and neuroprotective properties. It has appeared as a decent source for anti-anxiety and memory boosting. Rosemary contains terpenoids, essential oils, alkaloids, and flavonoids [246]. The most potent active compounds present in rosemary extracts are triterpenes, phenolic diterpenes and rosmarinic acid, carnosic acid, ursolic acid, and betulinic acid. Rosmarinic and carnosic acids have anti-inflammatory and antioxidants effects [247,248]. Aging and age-related diseases appear when the concentration of endogenous antioxidants is insufficient to eliminate free radicals. A study on mice has shown that a 60% extract containing carnosic acid for 90 days had a positive effect on memory of the tested animals. It was also shown that the extracts containing 20% carnosic acid improve cognitive impairment in the rat model, which may be the result of the antioxidant effect—a reduction in the area of hippocampus the levels of ROS, IL-1β, IL-6, and TNF-α. Another protective mechanism of rosemary extract is related to ability of carnosic acid to inhibit the activity of AChE and stimulating effect on BuChE in rats’ brain. It also decreases BuChE expression in the cortex and increased it in the hippocampus. This suggests that rosemary extract improves long-term memory by inhibiting AChE activity in the rat brain [246]. This is related to the theory that the release of ACh into the synaptic cleft is responsible for AD, due to inhibition of ACh hydrolysis. It causes degeneration of cholinergic neurons in the basal forebrain and is associated with the loss of cholinergic neurotransmission in the cerebral cortex. It is destructive, since the cholinergic system is involved in concentration and cognitive memory performance. Both forms of cholinesterase are present in the human brain in neurons, oligodendrocytes, astrocytes, and tangles and plaques in AD. AChE activity in AD is reduced in the cortex, and active BuChE remains unchanged or increases [246,249]. The antidepressant effect is demonstrated by the hydro-alcoholic extract from the rosemary leaves and stems (100 mg/kg p.o). In a behavioral study with the use of an animal model (mouse), administration of the extract has shown a similar effect to fluoxetine (10 mg/kg, per os) within 14 days. More research needs to be done to prove exactly which specific component of the extract is responsible for this effect. However, it is already known that the antidepressant effect of the extract is related to its influence on noradrenergic α-1; dopaminergic D1 and D2; and serotonergic 5-HT1A, 5-HT2A, and 5-HT3 receptors [250]. Another study revealed that the ursolic acid present in rosemary reduces the immobility period in the tail suspension test and in the FST in mice. The results of aforementioned animals’ tests suggest that the capacity of ursolic acid as antidepressant may be related to activation receptors D1 and D2 in dopaminergic pathway [251]. The influence of dopamine and the corresponding activity of the dopaminergic pathways has important role in the control of depression [252].

Theaflavins found in black tea inhibit the production of cytokines with pro-inflammatory properties, inhibit iĸB kinase and NF-ĸB activation as well as reduce dendrite atrophy in the prefrontal cortex and hippocampus, thereby improving cognitive abilities and alleviating depressive symptoms. The effects of theaflavins were more potent than catechins, chlorogenic acid, or caffeic acid. The potency was comparable to epigallocatechin-3-gallate (EGCG). It is not clear how theaflavins pass through the BBB. It is presumed that the barrier is damaged as a result of prolonged inflammation and thus allows theaflavin or monocytes affected by theaflavin to pass through [253].

*Petroselinum crispum* (parsley) belongs to the *Apiaceae* family and is a widely used culinary herb. The polyphenols found in parsley are apigenin, quercetin, luteolin, and kemferol. Parsley exhibits a number of beneficial effects, including antioxidant, analgesic, immunomodulatory, and spasmolytic. Moreover, mice studies have shown antidepressant and anxiolytic effects. Thus, parsley may prove to be an excellent antidepressant with fewer side effects than the synthetic drugs [254]. Catechin derivatives are found in abundance in green tea leaves, mainly (-)-epigallocatechin gallate (ECGC), (-)-epigallocatechin (EGC), (-)-epicatechin-3-gallate (ECG), and (-)-epicatechin. Both catechins and other polyphenols in green tea have the ability to chelate iron and copper ions, thereby inhibiting the formation of free radicals through the Fenton reaction. EGCG prevents the formation of Aβ fibrils and prevents lipid peroxidation by this protein. In addition, it has a protective effect on mitochondria and hippocampal cells by inhibiting caspase activation [255]. The L-theanine found in green tea exerts a number of beneficial effects. In addition to its antioxidant effects, it protects against the damaging impact of Aβ on cognitive function. Additionally, it may reduce stress by affecting serotonin and dopamine neurotransmission and inhibiting L-glutamic acid binding to the glutamate receptors [256]. Both ECGC and L-theanine have been shown to inhibit the ERK/p38 and NF-ĸB pathways, thereby preventing neuroinflammation [257,258]. Studies have shown that regular administration of green tea has antidepressant effects by increasing neurogenesis, as well as normalizing HPA axis function [259].

Polyphenols are secondary metabolites found in fruits, vegetables, wine, tea and oil. It is structurally diverse group, including flavonoids, phenolic acids, and lignans [176]. There are two major groups of polyphenols- flavonoids and non-flavonoids [260]. They are in part responsible for the French paradox, i.e., low incidence of cardiovascular diseases in Mediterranean populations with red wine and high saturated fat consumption [169]. Polyphenols are known for their antioxidant and anti-inflammatory properties. Many studies indicate the possible use of polyphenols of magnolia, green and black tea, and parsley, among others, in the prevention and treatment of AD and depression [253,254,255,256,257,258,259,261,262]. Salidroside (SAD) is a bioactive compound found in *Rhodiola rosea* L. It is used to treat AD and depression via antioxidative mechanism [253]. This polyphenolic component has: antifatigue, hypoglycemic, anti-inflammatory, antiperoxidation, and central inhibitory effect [263]. Due to imbalance between oxidation and antioxidation, there is an excess of ROS in cells, which indicates oxidative stress. Products like SOD, GPx, and CAT are antioxidant macromolecules [264,265]. *Rhodiola rosea* L. ethanol extract, contains a large amount of phenolic compounds, especially SAD, which has strong antioxidant activity in vivo and in vitro. SAD inhibits ROS production and also increasing the activity of endogenous antioxidant enzymes, GPx and SOD [266,267]. Resveratrol is a polyphenol found in high amounts in red grapes. It was decided to test its anti-inflammatory and antioxidant properties for depression. It has shown to reduce the level of markers closely related to depression. In order to investigate its effect on inhibiting the development of depressive, behaviors several different tests were used, such as the sucrose preference test or forced swimming. The sucrose preference test showed increased sucrose intake, counteracting the depressive effects associated with the reward system [268,269]. People suffering from depression also have a high level of cortisol. In preclinical studies, it was observed that the administration of resveratrol leads to a lower level of mentioned hormone in patients’ blood, compared to the control group. This is also done by adjusting the HPA axis [261,270,271]. As a polyphenolic compound, resveratrol has an antioxidant effect by reducing oxidative stress. Its administration leads to a decreased production of antioxidant enzymes and reduction in the level of ROS [253,262]. Moreover, depression leads to decreased production of BDNF. After the injection of resveratrol in the mice testes, an increase in its level was observed in the prefrontal cortex, amygdala, and hippocampus. Resveratrol appears to be a promising agent for treatment of depression with a good safety profile. Increased concentration of MDA and 8-OHdG in depression shows the existence of a clear relationship between the development of oxidative stress and depression. Administration of resveratrol at a dose of 15 mg/kg/day reduced the level of the above markers of inflammation [256,257,258]. The link between the depression and AD is confirmed by increased expression of TN-α following intracerebral infusion of Aβ. It is believed that the increased production of the inflammatory markers is due to the modification of the serotonin metabolism in the brain of people with depression. This is why mice with AD show decreased levels of serotonin in the brain and an increase in the concentration of its metabolites. Administration of the resveratrol reduces the formation of Aβ [254,259].

#### 6.3.2. Vitamin C

Vitamin C is a water-soluble 4,5-dihydroxyfuran-3-one with a 1,2-(dihydroxyethyl) substituent. Its pK1 is 4.2 and pK2 is 11.6, due to this the predominant form in the physiological pH of the body is AscH^−^. It is an excellent reductant that can undergo two successive one-electron oxidation processes to form ascorbic radical and dehydroascorbic acid. The ascorbic radical is relatively non-reactive, but can react with hydrogen ions to form AscH^−^ and DHA [158]. Some mammals (e.g., rats) are able to synthesize vitamin C for humans; however, it is an exogenous compound due to lack of enzyme (L-gulono-1,4-lactone oxidase) which is involved in production of this vitamin from glucose. The daily requirement for vitamin C is 75–90 mg [247]. Vitamin C plays several crucial roles in the nervous system. Its main function is neutralizing ROS formed in metabolic processes. However, no significant effect on RNS has been demonstrated at concentrations < 150μmol/L in plasma or extracellular fluid. Vitamin C shows this effect only at concentrations of 1–2.5 mmol/L in the cytosol of the cell [244]. In comparison, intracellular vitamin C levels range from 0.2 mmol/L to 10 mmol/L. In contrast, the plasma level of vitamin C in healthy person is 50–80 µmol/L [272]. Furthermore, to its antioxidant activity, it also acts as an enzymatic co-factor involved in the synthesis of many compounds such as tyrosine, collagen, peptide hormones, and carnitine. It also affects the synthesis and neurotransmission of catecholamines or participates in regeneration of vitamin E and glutathione. Alpha-tocopherol protects the cell membranes against lipid peroxidation. As a result of this process, α-tocopherol is oxidized to α-tocopheroxyl radical, which has a damaging potential. Vitamin C reduces the resulting radical, after which its oxidized form is reduced by NADH and NADPH [244]. Supplementation of the aged mice with both vitamin E and C has shown significant improvement of memory, while no beneficial antioxidant effect was obtained with only vitamin C supplementation [164,243]. Mice lacking vitamin C transporters have experienced extensive cranial hemorrhages and death on the first postnatal day [270].

In neurodegenerative diseases, such as Huntington’s disease (HD), PD, AD, and also psychiatric conditions, i.e., depression, anxiety, or schizophrenia, the decreased levels of cellular and plasma vitamin C and increased free radicals are observed, so their scavengers may exhibit therapeutic effects by inhibiting their negative effects. Ascorbic acid therapy has been shown to decrease cell death by reducing ROS and oxidative stress [158,244,268]. A study on 23 Alzheimer’s patients has shown that simultaneous supplementation with both vitamin E and C increased concentration of these vitamins in cerebrospinal fluid and showed antioxidant effects, while no significant impact on improving cognitive function was demonstrated [167]. However, it has been noted that vitamin C supplementation reduces the risk of AD [270]. Thus, it can be presumed that vitamin C plays an important role in the prevention of neurodegenerative diseases, but does not improve the patients’ condition.

## 7. Conclusions

Both chronic stress and oxidative stress are factors that play a significant role in the development of diseases related to neurodegenerative processes, including AD or depression. Many studies point to the important role of antioxidants in the prevention and treatment of these diseases. These substances are highly effective with low possibility of side effects. Among the compounds with antioxidants properties, the best results in treatment of AD should be prescribed to vitamin E, coenzyme Q10 melatonin, polyphenols, curcumin, and selenium. In the treatment of depression, by far the greatest potential has been observed for the antioxidants present in curcumin, zinc, selenium, vitamin E, and saffron. The wide range of many various properties of antioxidants means that these substances may act as an appropriate and effective treatments, treatment support, and prevention from the occurrence of neurodegenerative disorders.
